# Potent natural products and herbal medicines for treating liver fibrosis

**DOI:** 10.1186/s13020-015-0036-y

**Published:** 2015-04-15

**Authors:** Shao-Ru Chen, Xiu-Ping Chen, Jin-Jian Lu, Ying Wang, Yi-Tao Wang

**Affiliations:** State Key Laboratory of Quality Research in Chinese Medicine, and Institute of Chinese Medical Sciences, University of Macau, Macao, SAR China

## Abstract

Liver fibrosis is a wound-healing response to chronic liver injury characterized by progressive inflammation and deposition of extracellular matrix components. The pathological condition of liver fibrosis involves secretion of extracellular matrix proteins and formation of scar tissue. The major regulators involved in hepatic fibrogenesis are the transforming growth factor (TGF)-β1/SMAD and toll-like receptor 4 (TLR4)-initiated myeloid differentiation primary response 88 gene (MyD88)/NF-ĸB cell signaling pathways. This article reviews natural products and herbal medicines that have demonstrated activity against liver fibrosis through different mechanisms of action, including anti-hepatitis B and C virus activity, anti-inflammation, inhibition of cytokine production and nuclear receptor activation, and free radical scavenging.

## Introduction

Chronic liver injury increases extracellular matrix (ECM) deposition by activating the hepatic stellate cells (HSCs). This results in liver fibrosis, which is a major cause of mortality worldwide mainly because of chronic infection with the hepatitis virus and obesity associated with fatty liver disease [1]. Cirrhosis occurs in the final stage of liver fibrosis and is characterized by the distortion of liver vasculature and architecture that increases the likelihood of liver failure and primary liver cancer [2]. At present, there are no medications to manage liver fibrosis; the only treatment is tissue transplantation. The range of biological activities offered by natural products and herbal medicines has increased interest in their potential for treating liver fibrosis.

We review natural products and herbal medicines that have demonstrated activity against liver fibrosis through different mechanisms of action, including anti-hepatitis B and C virus activity, anti-inflammation, inhibition of cytokine production and nuclear receptor activation, and free radical scavenging. PubMed and Google Scholar were searched for references before the end of 2014 using the following combination of keywords: liver fibrosis and natural product; liver fibrosis and herbal medicine; liver fibrosis and Chinese medicine; liver fibrosis and Clinical trials; liver fibrosis and mechanism of action.

## Pathogenesis and molecular signaling pathways involved in liver fibrosis

### Pathogenesis of liver fibrosis

Liver fibrosis is a wound-healing response to chronic liver injury involving accumulated inflammation, which leads to the increased deposition of ECM and scar tissue [3]. It progresses at different rates in patients with various types of chronic liver injury [3]. Principal collagen-producing cells in the fibrotic liver include activated HSCs, portal fibroblasts, and myofibroblasts of bone marrow origin [4,5]. Among them, activated myofibroblasts are most responsible for forming fibrotic tissue associated with most chronic liver diseases [6]. The precise origin of activated myofibroblasts is unknown, but several types of cell may be implicated. Bone marrow-derived fibrocytes, or circulating mesenchyme cells, migrate through the injured liver and differentiate into myofibroblasts during fibrogenesis [7]. In addition, hepatocytes, sinusoidal endothelial cells, Kupffer cells, and lymphocytes may contribute to liver fibrosis [7].

Under normal conditions, HSCs store retinoid and remain in a quiescent state, with expression of adipocyte markers, including peroxisome proliferation-activated receptor-γ (PPAR-γ), sterol regulatory element binding protein-1c, and leptin [8]. HSCs are activated to produce different types of ECM proteins in conditions of chronic inflammation [9]. Activated HSCs are characterized by myogenic markers like α-smooth muscle actin (α-SMA), c-Myb, and myocyte enhancer factor-2 [10].

### TGF-β1 governs liver fibrosis

**Figure 1:**
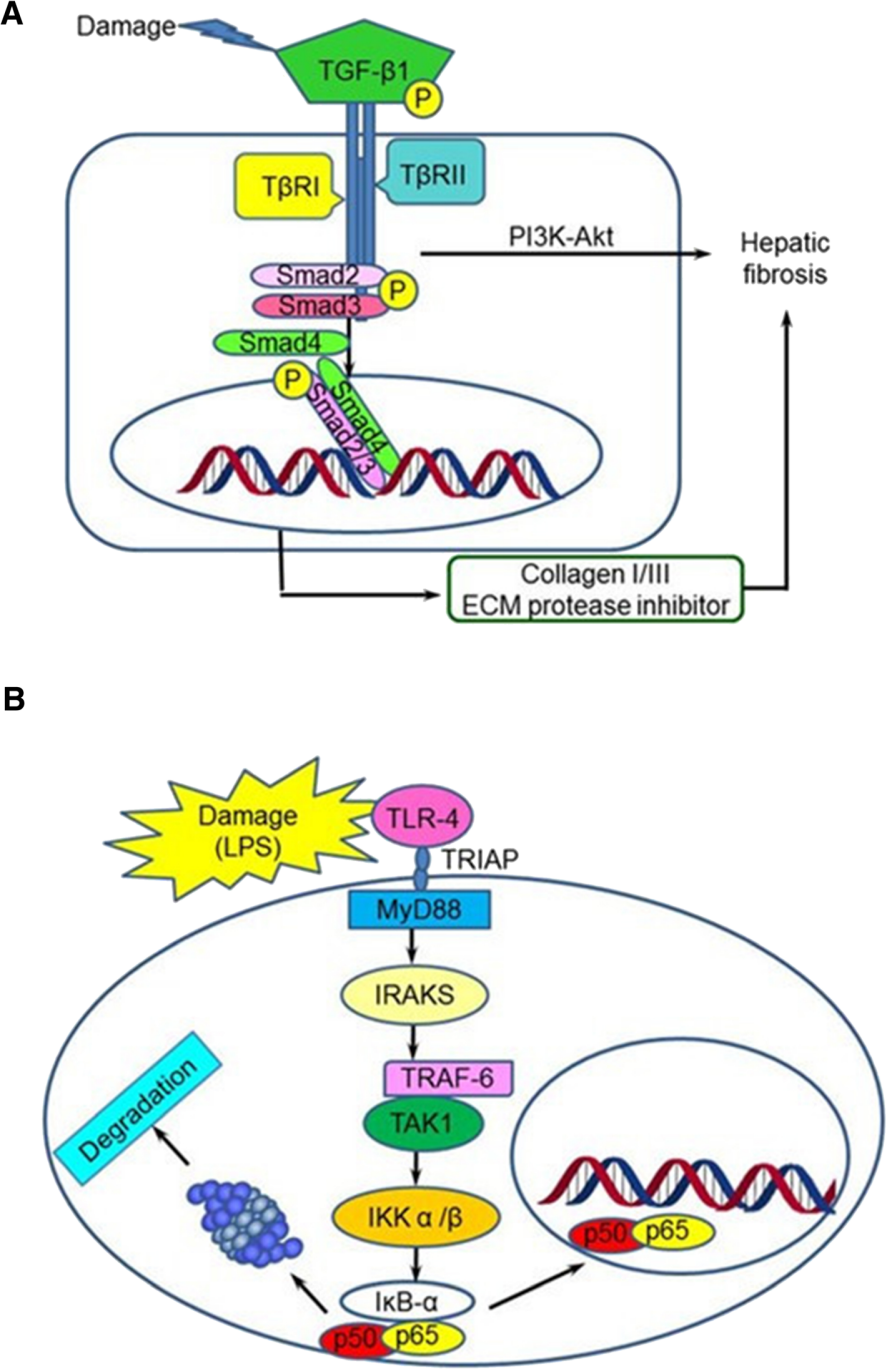
Signaling pathway mediates hepatic fibrogenesis: **(A)** the TGF-β1/SMAD signaling pathway, and **(B)** TLR4 activated-MyD88/TGFβ1/NFκB pathway.

TGF-β1 is a member of the TGF β superfamily [11] and is involved in liver fibrosis (Figure 1A). Under normal conditions, TGF-β1 binds to latency-associated peptide and remains inactivated. Upon activation, TGF-β1 binds to its receptors and phosphorylates the downstream signal SMAD2/3. Phosphorylated SMAD2/3 recruits the common mediator SMAD4 to form a hetero-oligomer complex. The SMAD complex then translocates into the nucleus and activates transcription of collagens [12]. Elevated collagen expression induces trans-differentiation of myofibroblasts, which secrete ECMS that can overwhelm the cellular capacity for ECM degradation and lead to fibrosis [13].

Sustained signaling from the TGF-β1 cascade proliferates HSCs, which also produce ECMs, resulting in fibrous scars [4]. TGF-β1 stimulates myofibroblast differentiation through the phosphatidylinositol 3-kinase (PI3K)-Akt pathway. Upon liver damage, TGF-β1 activates Akt signaling *via* p38 mitogen-activated protein kinase and focal adhesion kinase (FAK). Prolonged activation of the above cell signaling pathways ultimately leads to inflammatory conditions in the liver, resulting in liver fibrosis [14].

### TLR4 promotes liver fibrosis through transcription of inflammation cytokines

Almost all hepatic cells with elevated levels of TLR4 are related to fibrotic progression (Figure 1B) and promote liver fibrosis [15]. TLR4 induces transcription of genes related to fibrogenesis through the MyD88/NFκB cascade [16]. Upon damage, lipopolysaccharide (LPS) interacts with circulating LPS-binding protein and binds to TLR4 through the co-receptors cluster of differentiation 14 (CD14) and lymphocyte antigen 96 [17]. The LPS/TLR4 complex then activates downstream pathways *via* the bridging adaptor TIR-domain-containing adaptor protein (TRIAP) dependent on MyD88 or the TIR-domain-containing adapter-inducing interferon-β (TRIF).

In a MyD88-dependent manner, MyD88 recruits IRAK4 (IL-1 receptor associated-kinase-4) through an interaction between their death domains. Once activated, IRAK4 triggers further activation of IRAK1 and IRAK2. The activated IRAKs then dissociate from the MyD88 complex and interact with tumor necrosis factor receptor-associated factor-6 (TRAF6). The IRAKs/TRAF6 complex binds to TAK1 (TGF-β activated kinase 1), which subsequently leads to phosphorylation and ubiquitination of subunits of the IκB kinase complex. Then NF-κB will be released from the IκB kinase complex and translocate into the nucleus after IκBα phosphorylated and ubiquitinated [18], inducing transcription of inflammatory cytokines related to liver fibrogenesis, including interleukin 6 (IL-6), IL-12, and tumor necrosis factor α (TNFα) [19].

In the TRIF-dependent signaling pathway, LPS activates the receptors and recruits the adaptor TRIF. Subsequently, TRIF activates TANK-binding kinase 1 (TBK1) and receptor-interacting protein 1 (RIP1). The TRIF/TBK1 signaling complex then phosphorylates interferon (IFN) regulatory factor 3 (IRF3). The phosphorylated IRF3 translocates into the nucleus and activates transcription of type I IFN. The activated RIP1 also triggers polyubiquitination and activation of TAK1 and NF-κB.

### Bile acid homeostasis and xenobiotic detoxification receptors

Nuclear receptors and cell surface receptors regulate the initiation and progress of liver fibrosis [20]. Bile acid regulates hepatic lipid metabolism through binding to its nuclear receptor farnesoid X receptor (FXR) [21]. Bile acid-activated FXR induces transcription of repressor small heterodimer partner mRNA (SHP). SHP then binds to liver receptor homolog-1 and inhibits the expression of cholesterol 7α-hydroxylase (CYP_7_A_1_, or cytochrome P4507A1). Decreased CYP_7_A_1_ expression suppresses the multiple-step conversion of cholesterol to primary bile acid (or cholic acid) and thus attenuates the progression of liver fibrogenesis [22]. SHP also binds to retinoid X receptor α (RXRα) and retinoic acid receptor (RAR), which represses synthesis of the hepatic bile salt and thus reduces one of the contributing factors of liver fibrosis [23]. The activated FXR bound to SHP also promotes quiescence and apoptosis of activated HSCs in the fibrotic liver [24].

Bile acid also activates vitamin D receptor (VDR), a member of the nuclear hormone receptor superfamily [25]. Together with FXR, VDR regulates bile acid homeostasis and xenobiotic detoxification in the liver. VDR ligands prevent liver fibrosis by deactivation of HSCs through downregulation of the TGF-β1/SMAD pathway [25].

### Other cell signaling pathways involved in liver fibrosis

The FAK PI3K-Akt signaling pathway plays a major role in the activation of HSCs [26]. The PI3K complex consists of a p110 catalytic subunit and a p85 regulatory subunit. The activated PI3K catalyzes the reaction to produce Ptdlns(3,4,5)P3 and Ptdlns(3,4)P2, which bind to the pleckstrin homology domain of Akt and trigger its plasma membrane translocation and activation. Fully activated Akt promotes proliferation and survival in HSCs and other cell types [27].

The PPAR pathway is closely related to the activation of HSCs in liver fibrosis [28]. PPAR-γ, a key regulator of connective tissue homeostasis, inhibits fibrogenesis in HSCs and attenuates liver fibrosis *in vivo* [28]. The expression of PPAR-γ is mainly regulated by the TGF-β1/SMAD signaling pathway [29]. Other transcription factors like nuclear factor-like 2 (Nrf2) and cytokines (TNF-α, IL-6, IL-13) can also activate HSCs and promote liver fibrosis [30].

### Free radicals induce liver damage

Reactive oxygen species (ROS) are related to chronic liver damage and fibrogenesis [31]. ROS stimulate expression of cytokines, hormones, and growth factors and ROS-generated cytokines like platelet-derived growth factor (PDGF) and TGF-β1 are closely related to hepatic fibrogenesis [32].

### The balance of EMT and MET governs hepatic fibrogenesis

Epithelial-mesenchymal transition (EMT) is the process by which epithelial cells lose apicobasal polarity and intercellular adhesion complexes change phenotypes dramatically and move through the ECM like mesenchymal cells [33]. The balance between EMT and its reverse process, mesenchymal-to-epithelial transition (MET), determines the progression of liver fibrosis [33]. EMT is usually triggered by the growth factors-induced expression of Snail and TM4SF5 [34]. The activated HSCs in the fibrotic liver also undergo an EMT as revealed by elevated mesenchymal and epithelial markers [35]. Other regulators, such as cadherins, microRNAs, transcription factors (e.g., Pax, paraxis, and Fox) and growth factors (e.g., Wnts, FGFs, and ephrins) are also involved in maintaining the balance of EMT and MET [36,37].

### Commonly used liver fibrosis animal models

Laboratory animal models are based on the above pathological and molecular background of hepatic fibrosis. The most commonly used laboratory liver injury model is induced by chemical reagents like carbon tetrachloride (CCl_4_) or alcohol. Transgenic animal models manipulate the expression of key signaling molecules like TGF-β1 to mimic the pathological condition of liver fibrosis. We summarize the most commonly used animal models in Table 1.

**Table 1:** **Commonly used animal models in the study of liver fibrosis**

**Model**	**Inducing factor**	**Methodology**	**Characteristics**	**Reference**
Bridging fibrosis mice	CCl_4_	Eight-week-old male C57BL/6 J mice are intraperitoneally injected with 0.5-ml/kg body weight CCl_4_ (1:50 v/v in corn oil) or vehicle (DMSO in corn oil) three times a week for 4 weeks. Calcipotriol (20-μg/kg body weight) is administrated by oral gavage five times a week, commencing 20 days after the first dose of CCl_4_	Convenient, reproducible, well tolerated, most commonly used model	[115]
Alcohol-induced fibrosis	Alcohol	Alcohol in combination with Western diet is fed to mice intragastrically for 8 weeks	Aversion for alcohol, rapid metabolism, difficult to control fibrotic stage	[116]
Non-alcoholic steatohepatitis (NASH)-associated fibrosis	Methionine and Choline	Female C57BL/6 mice are fed a methionine-choline-deficient diet or a methionine-choline-supplemented diet for 10 weeks; the latter control diet is composed of MCD diet supplemented with L-methionine (1.7 g/kg) and choline bitartrate (14.48 g/kg)	Similar pathology to human NASH, well characterized, highly reproducible, lack of metabolic context, time consuming	[117]
Auto-immune fibrosis	Pig serum	Male Wistar rats are given intraperitoneal injections of 0.5-ml normal pig serum twice a week for 10 weeks with or without concomitant oral administration of PTX (20 mg/kg)	Mimics immunologic component, but lack of stability and time consuming	[118]
Biliary fibrosis	Bile duct ligation	Under methoxyflurane anesthesia, the common bile duct is double-ligated using 4–0 silk after a midline abdominal incision. Sham-operated mice have their common bile duct exposed and manipulated but not ligated	Reversible, but highly variable with high mortality rate	[119]
CRP-TGF-β1, IL-12p35^−/−^ dnTGFβR transgenic-genetic model	Overexpression of TGF-β1	Standard transgenic method	TGF-β1 susceptibility, pathophysiological significance, but expensive, and early death with limited application	[120]
Mdr2 (Abcb4^−/−^) transgenic-genetic model	Hepatobiliary phosphatidyl-choline	Standard transgenic method	Hepatic lesions resembling primary sclerosing cholangitis, convenient but expensive	[121]

## Potent natural products and herbal medicines for the management of liver fibrosis

From Table 2, we can see that there are a lot of potent natural products and herbal medicines for the management of liver fibrosis. But effective therapeutic options are limited in liver fibrosis [38]. Because of its diverse pathogenetic mechanisms, its management may include antiviral or anti-inflammatory approaches, inhibition of cytokine production, modulation of nuclear receptors, reduction of cellular oxidative stress, and EMT and MET balance.

**Table 2:** **Pharmacological effects of natural products and herbal medicines with anti-liver fibrosis activity**

**Natural product/herbal medicine**	**Pharmacological effect**	**Model system**	**References**
Helioxanthin	Inhibited HBV replication, suppressed IL-1-induced c-jun transcription and c-jun-mediated DNA binding activity of AP-1	HBV-producing HepG 2.2.15 cell line	[44]
Wogonin	Suppressed secretion of HBV antigens and reduced HBV DNA level through inhibition of HBV DNA polymerase activity	HBV-producing MS-G2 cell line	[46,47]
Matrine and oxymatrine	Inhibited HBV surface antigen secretion, E antigen, and HBV DNA replication	HBV-producing HepG 2.2.15 cell line	[48,49]
*Rhodiola kirilowii* Maxim	Inhibited HCV NS3 serine protease activity	Cos-7(NS3/4A-SEAP) cell line	[50]
Green tea	Inhibited HCV viral entry and replication	Primary human hepatocyte cells infected with HCV pseudoparticles, HCV-JFH1 viral culture system, patients with HCV infection and detectable viremia	[51-53]
Glycyrrhizin acid	Inhibited HCV full-length viral particle and HCV core gene expression syngenetically with IFNα, reduced hepatic inflammation, prevented apoptosis and inflammatory infiltrates	HCV-infected liver cells, BALB/c mice	[54-56]
Nobiletin	Inhibited HCV absorption, reduced hepatic inflammation	Human lymphoblastoid leukemia MOLT-4 cell line, and primary cultured rat hepatocytes	[57,58]
Genistein	Decreased levels of inflammation mediators, including IL-6, TNFα, and myeloperoxidase	CCl_4_-induced rat hepatic fibrosis	[61]
Salvianic acid A	Inhibited proliferation of HSCs, reduced expression of TGF-β1 and collagen I/III	HSC-T6 cell line	[62]
Betulin, betulinic acid	Inhibited expression of TNFα, TGF-β1, TIMP-1, TIMP-2, and MMP-2	Alcohol-induced liver fibrosis	[63,64]
*Gexia Zhuyu Tang*	Attenuated fibrogenesis and reduced inflammation, reduced CCl_4_-induced collagen deposition	Late-stage liver fibrosis patients, CC_4_-induced mouse liver fibrosis	[65,66]
*Yanggan Wan*	Deactivated HSCs through epigenetic de-repression of PPAR-γ	Bile duct-induced cholestatic mouse liver fibrosis	[71]
Rosmarinic acid, baicalin	De-repressed PPAR-γ through suppression of canonical Wnt signaling in activated HSCs	Bile duct-induced cholestatic mouse liver fibrosis	[71]
*Yin Chen Hao Tang*	Decreased serum IFN-γ and IL-12 levels, inhibited α-SMA activation and transcription of its target genes	Rat liver fibrosis model	[75,76]
Paeoniflorin	Reduced the size of egg granuloma, fibrosis scores, serum IL-13 levels, and hydroxyproline content, and blocked IL-13 signaling pathway	CCl_4_-induced rat hepatic fibrosis	[77,78]
Oleanolic acid, ursolic acid	Inhibited bile acid production by blocking the interaction between FXR and its coactivator SRC-3 and endogenous ligand chenodeoxycholic acid, suppressed expression of FXR-targeted bile salt export protein, reduced hepatic free radicals through increasing hepatic transcription of Nrf2 target genes	HepG2 cell line, wild-type and Nrf2-null mice	[80-82]
Silymarin	Protected liver from further damage through antioxidant and anti-inflammatory activity	CCl_4_-induced rat liver fibrosis	[84,85]
Silybinin	Inhibited TGF-β1-induced collagen secretion and oxidase stress	Thioacetamide-induced rat liver fibrosis	[86]
Acanthus ilicifolius alkaloid A	Reduced lipid peroxidation and oxidative stress	CCl_4_-induced mouse liver fibrosis	[87,88]
Curcumin	Suppressed multiple proangiogenic factors that modulate cannabinoid receptors, inhibited ECM expression, decreased collagen deposition, increased serum MMP-13 and glutathione levels	CCl4-induced rat liver fibrosis	[89-91]
β-caryophyllene	Exhibited high scavenging activity against hydroxyl radicals and superoxide anions, inhibited lipid peroxidation, suppressed expression of Col1a1 and TIMP-1	CCl_4_-induced mouse liver fibrosis	[93]
*Diwu Yanggan* formula	Modulated the EMT and MET balance	CCl_4_-induced rat liver fibrosis	[94]
Salvianolic acid B	Abrogated EMT-induced fibrogenesis	Renal fibrosis model	[96]
*Fuzheng Huayu* tablet	Reversed EMT in the fibrotic kidney through suppression of α-SMA, TGF-β1, and nuclear translocation of SMAD3; induced apoptosis through p38 and SAPK/JNK pathways; decreased transcription of TIMP-1, PDGF-B, and PDGF receptor β1; reversed HBV-induced fibrosis and cirrhosis; prevented TGF-β1-induced EMT	Renal fibrosis model, HSC-T6 cells, patients with chronic hepatitis B, patients with cirrhosis caused by hepatitis B, renal fibrosis rats	[95,97-105,122,123]
*Fufang Biejia Ruangan* pill	Downregulated TGF-β1/SMAD pathway transduction	Rats, HSC-T6 cell line, and clinical trial	[45,49]
Silybin-phospholipids and vitamin E complex	Reduced liver fibrosis scores and downregulated fibrosis markers, deactivated HSCs and downregulated TGF-β1 and TNFα	Fatty liver-associated HCV-positive patients	[109,110]
Obeticholic acid	Inhibited synthesis and accumulation of bile acid in the liver, reduced liver inflammation and fibrosis	Patients with type 2 diabetes and non-alcoholic fatty liver disease	[111,112]
Docosahexaenoic acid	Suppressed Procol1α1 and TGF-β1, inhibited hepatic inflammation and oxidative stress markers, including TLR4, TLR9, CD14, MyD88, and NADPH oxidase subunits	Western diet-induced NASH in Ldlr(^−/−^) mice	[114]

### Antiviral drugs attenuate chronic hepatitis infection

Chronic hepatitis B and C virus (HBV and HCV) infection is the leading cause of liver fibrosis. Nucleoside/nucleotide analogs like Entecavir or Lamivudine are used to treat HBV infection [39-41]. Peginterferon α plus ribavirin and direct acting antivirals are used to treat HCV patients [42]. Long-term use of most virus-targeted antiviral therapies may cause drug resistance and side effects, so agents to illuminate drug resistance are needed.

**Figure 2:**
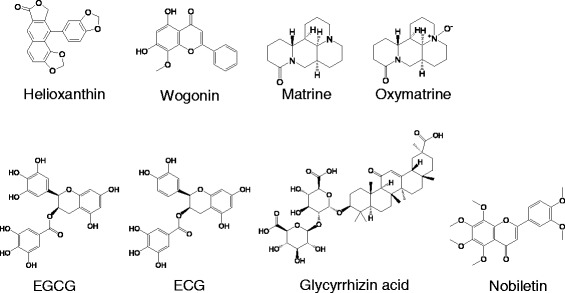
Natural products that inhibit HBV, and HCV replication.

Helioxanthin (Figure 2) was originally isolated from the shrub *Taiwania cryptomerioides* (*Taiwan Shan*). Helioxanthin exhibited potent inhibitory activity against HBV replication in HepG 2.2.15 cells and Lamivudine-resistant HBV L536M/M550V double mutant HBV strain [43]. Treatment with helioxanthin suppressed IL-1-induced c-jun transcription and c-jun-mediated DNA-binding activity of AP-1 [44]. In one study, a synthesized derivative of helioxanthin, 8–1, suppressed HBV replication by decreasing the binding of hepatocyte nuclear factors 3 and 4 to the HBV replication machinery [45].

The herbal medicine *Scutellaria baicalensis* (*Huangqin*) has been used to reduce inflammation [46]. Wogonin (Figure 2) isolated from *Scutellaria baicalensis* exhibited potent anti-HBV activity both *in vitro and in vivo*. Treatment with wogonin suppressed secretion of HBV antigens and reduced HBV DNA levels through inhibition of HBV DNA polymerase activity in the HBV-producing MS-G2 cell line [47].

Matrine and oxymatrine (Figure 2), two alkaloids isolated from the root of the plant *Sophora japonica* (*Kushen*), reversed liver fibrosis through downregulation of the TGF-β1 pathway [48]. A combination treatment of matrine or oxymatrine with Lamivudine reduced chronic HBV infection-induced liver fibrosis through inhibiting secretion of hepatitis B surface antigen (HBsAg) and hepatitis B e antigen (HBeAg), and replication of HBV DNA [49].

Polyphenols (−)-epigallocatechin-3-O-gallate (EGCG) and (−)-epicatechin-3-O-gallate (ECG) (Figure 2), from the herbal medicine *Rhodiola kirilowii* (Regel) Maxim (*Xiaye Hongjingtian*) exhibited potent inhibitory effect against HCV NS3 serine protease with low cytotoxicity [50]. Polyphenols extracted from green tea also exhibited potent activity against HCV viral entry [51] and replication [52]. In one study, a single oral administration of green tea extract containing 94% pure EGCG was safe and well-tolerated by all 11 patients with cirrhosis associated with chronic HCV infection [53].

Glycyrrhizin acid (Figure 2), the major component of the root of *Glycyrrhiza glabra* (*Yanggancao*), inhibited expression of the HCV full-length viral particle and HCV core gene synergistically with IFNα [54]. Treatment with glycyrrhizin acid reduced hepatic inflammation through regulation of CD4^+^ T cell response in a JNK-, ERK-, and PI3K-Akt-dependent manner in mice with liver injury [55]. Glycyrrhizin acid prevented apoptosis and inflammatory infiltrates induced by LPS/GaIN injection through disturbing the binding of HMGB1 protein to the promoter of Gsto1 [56].

Nobiletin (3′,4′,5,6,7,8-hexamethoxyflavone, Figure 2), the active component of *Citrus unshiu* peel, markedly inhibited HCV absorption in the human lymphoblastic leukemia MOLT-4 cell line [57] and reduced hepatic inflammation through reducing iNOS and DNA-binding activity of nucleus NF-κB [58].

### Anti-inflammatory drugs reduce liver inflammation

Anti-inflammatory drugs, including corticosteroid, prednisone, and prednisolone, suppress cytokine transcription, thus inhibiting hepatic collagen deposition [59]. These drugs can only effectively and safely treat liver fibrosis in combination with azathioprine [59].

**Figure 3:**
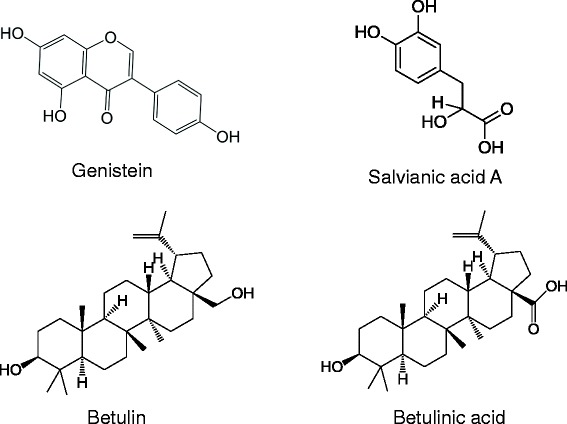
Chemical structures of natural products with anti-inflammation activity, including genistein, salvianic acid A, botulin, and betulinic acid.

Genistein (Figure 3) is a type of isoflavone first isolated from *Hydrocotyle sibthorpioides* (*Tianhusui*) and considered a potent chemopreventive agent with estrogenic activities against breast cancer [60]. Treatment with genistein decreased levels of inflammation mediators, including IL-6, TNF-α, and myeloperoxidase, through downregulation of NF-κB in alcohol- and CCl_4_-induced liver fibrosis in rats [61].

Salvianic acid A (Figure 3), one of the most widely used natural products in China, is the main active component of *Salvia miltiorrhiza* (*Danshen*). In one study, treatment with salvianic acid A inhibited proliferation of HSCs and thus reduced the expression of TGF-β1 and collagen I/III. Decreased TGF-β1 levels led to inhibition of plasminogen activator, upregulation of the urokinase-type plasminogen activator, and dephosphorylation of Akt and ERK1/2 [62].

Betulin and its oxidized form betulinic acid (Figure 3), a type of triterpene derived from the bark of *Betula platyphylla var. japonica*, can reverse alcohol-induced cytotoxicity in HepG2 cells [63]. Treatment with betulin and betulinic acid inhibited expression of TNF-α, TGF-β1, tissue inhibitor of metalloproteinase (TIMP)-1, TIMP-2, and activated matrix metalloproteinase (MMP)-2 in alcohol-induced liver fibrosis *in vivo* [64].

The Chinese herbal formula, *Gexia Zhuyu* decoction (*Gexia Zhuyu Tang*; GZT), can attenuate fibrosis and reduce inflammation in the late stage of liver fibrosis [65]. Treatment with GZT improved degeneration and inflammatory necrosis in liver cells, and reduced CCl_4_-induced collagen deposition *in vivo* [66].

### Cytokine inhibitors attenuate hepatic fibrogenesis

Research shows that suppression of insulin-like growth factor I improves liver function and reduces liver fibrosis through upregulation of MMPs and downregulation of TIMPs [67]. Elevated levels of IL-17 and its receptors in response to liver damage may also promote the production of IL-1, IL-6, TGF-α, and collagen I that promote liver fibrosis. Treatment of these kinds of cytokine inhibitors could contribute to inflammation accumulation in the fibrotic liver [68]. The cytokine inhibitors can also decrease IL-22 expression-induced HSC senescence and inhibit hepatic fibrogenesis [69].

**Figure 4:**
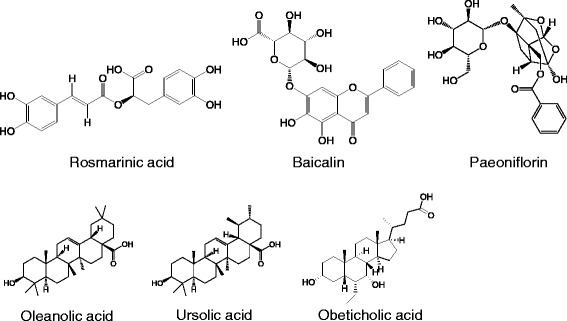
Cytokine inhibitors, including ursolic acid, paeniflorin, baicalin, and rosmarinic acid, and nuclear receptor modulators, including oleanolic acid, ursolic acid, and obeticholic acid as nuclear receptor modulators.

The herbal formula *Yanggan Wan* (YGW) has been shown to be hepatoprotective [70], deactivating HSCs through epigenetic de-repression of PPAR-γ in common bile duct-induced cholestatic liver fibrosis mice [71]. The de-repressed PPAR-γ was induced by reduced MeCP2 expression and its recruitment to the PPAR-γ promoter [71]. Rosmarinic acid and baicalin (Figure 4) from YGW de-repressed PPAR-γ through suppression of canonical Wnt signaling in activated HSCs [71]. Baicalin shifts the balance of profibrotic to antifibrotic cytokines and reduces oxidative stress in the fibrotic liver in experimental animal models [72], while romarinic acid can also inhibit proliferation and induce apoptosis of HSCs [73].

Treatment with *Yin Chen Hao* decoction (*Yin Chen Hao Tang*; YCHT) can decrease serum IFN-γ and IL-12 levels [74]. In the DMN-induced liver fibrotic rat, treatment with YCHT significantly improved the pathological condition through inhibition of α-SMA activation and transcription of its target genes [75].

Paeoniflorin (Figure 4), the major bioactive constituent of *Moutan cortex* (*Mudanpi*), effectively attenuated CCl_4_-induced liver fibrosis [76]. In one study, treatment with paeoniflorin reduced the size of egg granuloma, fibrosis scores, IL-13 serum concentration, and hydroxyproline content in the liver of mice infected with *S. japonicum*. Paeoniflorin also showed an inhibitory effect on hepatic fibrogenesis through downregulation of IL-13 expression and abrogation of the IL-13 signaling pathway in activated HSCs [77].

### Nuclear receptors that modulate liver fibrogenesis

FXR is highly expressed in activated HSCs in the fibrotic liver [78]. FXR ligands prevent hepatic fibrogenesis through deactivation of HSCs and decreased ECM expression [78].

Oleanolic acid and ursolic acid (Figure 4) are triterpenoid saponins commonly identified in medicinal plants [79]. Treatment with oleanolic acid inhibited the production of bile acids through blocking the interactions between FXR and its coactivator SRC-3 and endogenous ligand chenodeoxycholic acid [80]. Oleanolic acid blocked the binding of endogenous ligand chenodeoxycholic acid to FXR, suppressing expression of FXR-targeted bile salt export protein. There is evidence that oleanolic acid and ursolic acid reduce hepatic free radical content through increasing hepatic transcription of Nrf2 target genes, including NAD(P)H:quinone oxidoreductase 1, glutamate-cysteine ligase, catalytic subunit, heme oxygenase-1, and Nrf2 itself [81,82].

### Antioxidants as hepatoprotective agents in liver fibrosis management

**Figure 5:**
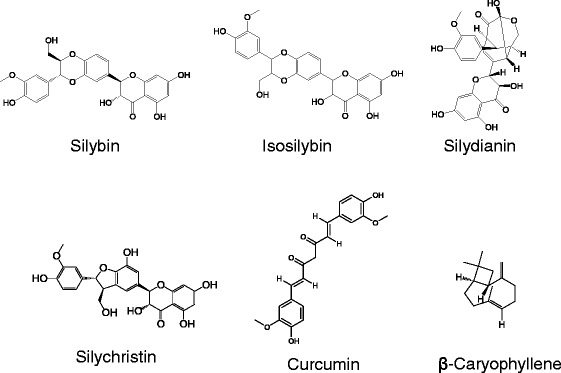
Chemical structures of antioxidative agents, including silybin, isosilybin, silydianin, silychristin, curucmin, and β-caryophyllene.

Silymarin, the extract of the milk thistle or *Silybum marianum* (*Shuifeiji*), consists of four flavonolignan isomers: silybin, isosilybin, silydianin, and silychristin (Figure 5). Silymarin has been widely used as a single-herb remedy for treating liver diseases. Silymarin treatment protects further liver damage by its antioxidant and anti-inflammatory activities [83,84]. Silybinin, also called silybin, exhibited hepatoprotective and antifibrogenic effects by inhibiting TGF-β1-induced collagen secretion and oxidase stress both *in vivo* and *in vitro* [85].

Acanthus ilicifolius alkaloid A (4-hydroxy-2(3H)benzoxazolone) was first isolated from *Acanthus ilicifolius* (*Juechuangle*) [86]. Acanthus ilicifolius alkaloid A and its acyl derivatives have been used as antioxidant, anti-inflammatory, and analgesic agents [87]. This group of analogs inhibits progression of CCl_4_-induced liver fibrosis by reducing lipid peroxidation and oxidative stress [88].

Curcumin (Figure 5), the main active component of turmeric, protects the CCl_4_-induced fibrotic liver through its antioxidant activity [89,90]. Treatment with curcumin suppressed multiple pro-angiogenic factors that modulate cannabinoid receptors and inhibited ECM expression, thus decreasing collagen deposition [89,91]. In one study, treatment with curcumin also increased serum MMP-13 and glutathione levels, thus reversing fibrosis in CCl_4_-induced liver fibrosis in rats [91].

β-caryophyllene (Figure 5) is a natural sesquiterpene identified in the essential oil of numerous plants and fruits [92]. In one study, β-caryophyllene exhibited high scavenging activity against hydroxyl radicals and superoxide anions, which inhibited lipid peroxidation and suppressed expression of Col1a1 and TIMP-1 in CCl_4_-induced mouse liver fibrosis [93].

### Modulation of the balance between EMT and MET to treat liver fibrosis

Treatment with *Diwu Yanggan* (DWYG) significantly decreased the hepatic hydroxyproline content and degree of CCl_4_-induced liver fibrosis in rats [94] and stimulated MET in the fibrotic liver through inhibition of the TGF-β1/BMP-7 signaling pathway [94].

Research shows that *Fuzheng Huayu* (FZHY) formula reverses EMT in the fibrotic kidney through suppression of α-SMA, TGF-β1, and nuclear translocation of SMAD3 [95]. In one study using the renal fibrosis model, treatment with salvianolic acid B from *Danshen* abrogated EMT by counteracting the TGF-β1 signaling pathway [96].

### Herbal medicines and natural products in clinical trials

FZHY formula to treat liver fibrosis [97] has completed phase II clinical trials approved by the FDA [98,99]. FZHY tablet consists of *R. miltiorrhizae* (*Danshen*), fermented *Mycelium* (*Chongcao*), *Semen persicae* (*Taoren*), *Fructus schisandrae* (*Wuweizi*), *Chinensis pollen pini* (*Songhuafen*), and *Gynostemma pentaphyllammak* (*Jiaogulan*) [100]. Administration of FZHY tablet improved liver function, serum fibrotic parameters and cirrhosis; decreased portal pressure; and regulated immune function and amino acid balance in 216 liver fibrosis patients with chronic HBV in a multicenter study [98]. Patients with posthepatitic cirrhosis showed improved liver function, decreased fibrotic score, prolonged 2-year survival, and reduced symptom scores after administration of FZHY tablet [101]. A multicenter, double-blind, randomized, and controlled clinical trial confirmed the efficacy of FZHY tablet, which decreased serum hyaluronic acid levels in patients with chronic HBV caused by cirrhosis [102]. Supplemental administration of FZHY tablet with nucleos(t)ide analogs to patients with chronic HBV also decreased serum fibrosis markers, including hyaluronic acid, laminin, amino-terminal propeptide of type III procollagen, and IV collagen [103].

FZHY tablet exhibits multiple mechanisms of action against liver fibrosis/cirrhosis [101]; its known active ingredients include salvianoic acid B and adenosine [102]. FZHY tablet exhibits antifibrotic activity by inducing apoptosis in HSC-T6 cells through p38 and SAPK/JNK pathways [99,104], and inhibits liver fibrosis through decreasing transcription of TIMP-1, PDGF-B, and PDGF receptor β1 *in vivo* [99]. A study of chronic HBV infection-induced fibrosis and cirrhosis patients showed that its therapeutic efficacy is closely related to the GA plus AA polymorphism of CYP_1_A_2_ [105].

*Fufang Biejia Ruangan* (FFBJRG) pill, consisting of *Carapax Trionycis* (*Biejia*), *Radix Paeoniae Rubra* (*Chishao*), *Radix Angelicae Sinensis* (*Danggui*), *Codonopsis Pilosula* (*Dangshen*), and *Radix Astragali* (*Huangqi*), is the first anti-liver fibrosis drug approved by the China Food and Drug Administration [106]. FFBJRG pill inhibited hepatic fibrosis *in vitro* and *in vivo* by inhibiting TGF-β1/SMAD pathway transduction [107] and is currently in phase IV clinical trials in the United States for the treatment of chronic HBV infection-associated liver fibrosis [108].

Treatment with silybin-phospholipids and vitamin E complex (SPV complex) significantly reduced liver fibrosis scores and downregulated fibrosis markers in fatty liver-associated HCV-positive patients in 11 Italian and 2 Romanian centers [109]. The SPV complex is currently in phase III clinical trials in the US for the treatment of liver fibrosis. The anti-liver fibrosis effect of the SPV complex is mainly due to deactivation of HSCs and downregulation of TGF-β1 and TNF-α expression [110].

**Figure 6:**
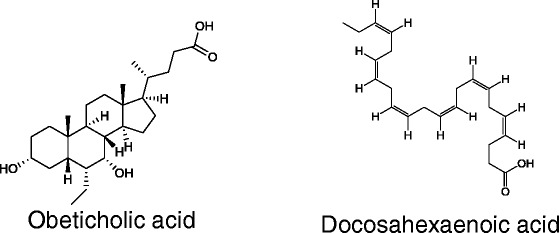
Chemical structures of oleanolic acid, and docosahexaenoic acid.

Obeticholic acid (Figure 6) is a semisynthetic derivative of bile acid and an FXR agonist. Treatment with obeticholic acid inhibits synthesis and accumulation of bile acid in the liver [111]. Patients with type 2 diabetes and non-alcoholic fatty liver disease showed significantly reduced markers of liver inflammation and fibrosis after administration of 25 mg obeticholic acid for 6 weeks in a phase II clinical trial in the US [112].

Docosahexaenoic acid (DHA, Figure 6), an omega-3 fatty acid, was originally isolated from maternal milk or fish oil. DHA is in phase II clinical trials for the treatment of liver fibrosis in the US [113]. Treatment with DHA inhibited hepatic fibrogenesis through suppression of Procol1α1 and TGF-β1 and inhibited hepatic inflammation and oxidative stress markers, including TLR4, TLR9, CD14, MyD88, and different NADPH oxidase subunits [114].

## Conclusion

Despite cumulative evidence of success in treating liver fibrosis, *in vivo* results are insufficient to confirm the clinical efficacy of natural products and herbal medicines for liver fibrosis. The identification of resources and the molecular mechanisms of action of these substances remain extremely challenging.

## Abbreviations

CCl_4_: Carbon tetrachloride
ECM: Extracellular matrix
EMT: Epithelial-mesenchymal transition
FOX: Forkhead box
FXR: Farnesoid X receptor
HBV: Hepatitis B virus
HCV: Hepatitis C virus
HSC: Hepatic stellate cell
IFN: Interferon
IL: Interleukin
IRAK: Interleukin-1 receptor-associated kinase
LPS: Lipopolysaccharide
MET: Mesenchymal-epitelial transition
MMP: Matrix metalloproteinase
MyD88: Myeloid differentiation primary response gene 88
Nrf2: Nuclear factor (erythroid-derived 2)-like 2
PDGF: Platelet-derived growth factor
PI3K: Phosphatidyl inositol 3-kinase
PPAR: Peroxisome proliferator-activated receptor
ROS: Reactive oxygen species
SHP: Small heterodimer partner mRNA
α-SMA: α-smooth muscle actin
TAK1: TGF-β activated kinase 1
TIMP: Tissue inhibitor of metalloproteinase
TLR: Toll-like receptor
TNF: Tumor necrosis factor
TRAF6: Tumor necrosis factor receptor-associated factor-6
VDR: Vitamin D receptor
